# Identifying Provider Prescribing Practices for GLP‐1RAs and SGLT‐2is in Patients With Type 2 Diabetes to Address Pharmacoinequity

**DOI:** 10.1155/jdr/7439681

**Published:** 2026-02-26

**Authors:** Parnnate Wongsirisakul, Vincent L. Chen, Ponni V. Perumalswami

**Affiliations:** ^1^ Division of Gastroenterology and Hepatology, University of Michigan, Ann Arbor, Michigan, USA, umich.edu

**Keywords:** GLP-1RA, liver disease, pharmacoequity, provider interview, SGLT-2i, Type 2 diabetes

## Abstract

**Background and Aims:**

Evidence‐based treatment of metabolic dysfunction‐associated steatotic liver disease (MASLD) in patients with Type 2 diabetes (T2DM) focuses on glycemic control and lifestyle modification to achieve weight loss, which slows the progression of liver disease and reduces the risk of liver‐related complications. We aimed to qualitatively explore provider‐level determinants in glucagon‐like peptide‐1 receptor agonists (GLP‐1RAs) and sodium/glucose cotransporter 2 inhibitors (SGLT‐2is) prescription.

**Methods:**

We identified a cohort of 189 outpatient adult primary care providers who cared for at least 50 annual T2DM patients in a tertiary academic health system in the United States in 2021. GLP‐1RA and SGLT‐2i prescribing rates were calculated with a median rate of 0.494 (IQR 0.393–0.594). Guided by the Consolidate Framework for Implementation Research, we conducted 1:1 interviews with high‐prescribing and low‐prescribing providers. All providers interviewed were either MDs or DOs.

**Results:**

Nine providers from the high‐prescribing quartile (prescribing rate 59.5%–78.6%) and eight from the low‐prescribing quartile (prescribing rate 4.8%–39.3%) were interviewed. High‐prescribing providers reported more patient‐facing hours and exposure to GLP‐1RAs and SGLT‐2is in residency. All providers cited task‐sharing, patient–provider relationship longevity, and medication effectiveness as facilitators of GLP‐1RA and SGLT‐2i prescription. Barriers to prescribing included medication costs, prior authorization processes, and prescription inertia. Using the Expert Recommendations for Implementing Change, we mapped barriers and facilitators to implementation strategies.

**Conclusions:**

This interview series identified key barriers and facilitators of GLP‐1RA and SGTL‐2i prescription. Implementation strategies may help improve prescribing highly effective, newer treatments in patients with T2DM, including those with metabolic comorbidities.

## 1. Introduction

Metabolic dysfunction‐associated steatotic liver disease (MASLD) can lead to liver failure and liver cancer, and is a major, growing cause of death in people with Type 2 diabetes (T2DM) [1]. Evidence‐based treatment of MASLD in T2DM focuses on glycemic control and lifestyle modification to achieve weight loss as this has been shown to slow progression of liver disease and reduce the risk of liver‐related complications. In the past 20 years, two newer classes of T2DM medications became available in the United States (U.S.)—glucagon‐like peptide receptor agonists (GLP‐1RAs) and sodium/glucose cotransporter 2 inhibitors (SGLT‐2is). These newer T2DM medication classes have a lower risk of hypoglycemia compared with sulfonylureas or insulin, induce weight loss, and have significant cardiovascular and renal benefits [2]. Although dipeptidyl peptidase‐4 inhibitors (DPP‐4is) were also released during this time, given the studies on pharmacoinequity of SGLT‐2is and GLP‐1RAs, this study focused on these two agents.

Treatment disparities and social determinants of health (SDOH) have important implications for people with T2DM. Prior research in a range of populations has observed racial and ethnic disparities in prescription rates of GLP‐1RA and SGLT‐2i agents despite controlling for high cost‐sharing based on health care coverage [3, 4]. Pharmacoequity [5–7] is central to overcoming health disparities and optimizing care for patients. Racial and ethnic minority individuals have been less likely than White persons to be prescribed novel guideline recommended therapies with proven effectiveness. These disparities may be particularly salient among patients with T2DM because Asian, Black, and Hispanic or Latino persons have a higher prevalence of diabetes and its complications than White patients [8]. Provider‐level barriers and facilitators of GLP‐1RA and SGLT‐2i use in T2DM have not been adequately researched. In this study, we aim to qualitatively explore provider‐level barriers and facilitators in GLP‐1RA and SGLT‐2i prescribing practices. Our interviews included topics in patient SDOH, provider collaboration, and general T2DM treatment approaches.

## 2. Materials and Methods

### 2.1. Eligibility

We identified a contemporary cohort of 189 outpatient adult primary care providers (PCPs) who have cared for at least 50 annual T2DM patients at an academic, tertiary care health system (Michigan Medicine) for the study period of January 1, 2021 to December 31, 2021. To estimate SGLT‐2i and GLP‐1RA prescribing rates, we included patients who met the following criteria: (1) have a T2DM diagnosis code, (2) are adults (≥ 18 years), and (3) had at least one outpatient clinical encounter with a Michigan Medicine PCP during the study period. We then evaluated the number of eligible T2DM patients who started on SGLT‐2i and GLP‐1RA during the study period. To determine prescribing rates, the number of patients prescribed a GLP‐1RA and/or an SGLT‐2i was divided by the total number of T2DM patients a provider received during the study period. Afterward, prescribing rates were categorized into quartiles.

A lifetime lookback period was used for exclusion criterion. We excluded patients with contraindications to either or both SGLT‐2i or GLP‐1RA: (1) patients with estimated glomerular filtration rate < 30 mL/min/1.73m^2^; (2) diagnosis codes for end stage renal disease, chronic kidney disease Stage 4 or 5, or dialysis at any time point in the study period; (3) history of lower limb amputation; (4) history of diabetic ketoacidosis (for SGLT‐2i); (5) personal history of medullary thyroid cancer, multiple endocrine neoplasia Type 2, or pancreatitis (for GLP‐1RA); and (6) history of allergy/intolerance to GLP‐1RA and/or SGLT‐2i. Pregnancy was not included as a condition for exclusion as it is an episodic event that we did not anticipate significantly changing the prescription rate of GLP‐1RAs and/or SGLT‐2is. Eligible providers have worked at Michigan Medicine for the entire study period and were licensed medical providers (MD, DO, NP, or PA‐C). Trainee providers such as residents and fellows were also excluded.

### 2.2. Ethical Compliance

All research activities were approved and reviewed by the University of Michigan Institutional Review Board prior to study initiation.

### 2.3. Data Collection

Providers from the high and low‐prescribing quartiles were invited to participate in 1:1 semi‐structured interviews (see Supporting Information 2 for the interview guide). The final number of interviews was determined based on saturation achievement when no new themes emerged. The interviews were 30 min in length, conducted virtually in 2025, and audiotaped. Participants were provided with a $100 incentive for study participation during off‐duty hours. The Consolidated Framework for Implementation Research (CFIR) [9, 10] functioned as a conceptual framework to study pharmacotherapy prescribing behavior and was used to develop the interview guide. Questions from each CFIR construct were adapted to fit study needs. The CFIR model was chosen because it is ideal for capturing contextual (inner and outer), individual, and innovative factors that have the potential to influence prescribing patterns. Inner contextual factors refer to features within the organization where the intervention is being implemented such as workplace culture and leadership engagement. Outer contextual factors refer to features of the broader environment such as external policies and patient preferences.

### 2.4. Analysis

Interview transcripts were autotranscribed and manually reviewed by our research assistant using individual audio recordings for accuracy. Transcripts were then reviewed by an analyst trained in qualitative research methods (P.W.) with oversight by senior study staff (P.V.P.). Analyses were completed using a rapid data analysis process—a qualitative approach used with time‐sensitive, targeted, and actionable qualitative information [11]—to group the apparent themes according to the National Institute of Minority Health and Health Disparities [12] and the Tailored Implementation for Chronic Disease (TICD) checklist [13]. The TICD has 57 individual determinants grouped in seven domains: (1) intervention factors, (2) individual health professional factors (which include (A) knowledge and skills, (B) cognition and attitudes and (C) professional behavior), (3) client/patient factors, (4) professional interactions, (5) incentives and resources, (6) capacity for organizational change, and (7) social, political, and legal factors. Because the TICD checklist was developed for patients with chronic health conditions, such as NAFLD and T2DM, and strikes a balance between individual‐level factors and health behavior change theories, it was ideal for this study. Provider‐prescribing barriers and facilitators were then mapped to implementation strategies based on the evidence‐based Expert Recommendations for Implementing Change (ERIC) nomenclature [14] so that they can be considered for testing in a future trial to promote provider behavior change.

## 3. Results

On average, high‐prescribing providers had more patient‐facing hours per week (25 vs. 21.5), and low‐prescribing providers had 10.5 more years of experience working as medical faculty (standard deviation 7.8). A table summarizing interview participant details is included below (see Table 1). When asked, all providers denied that the COVID‐19 pandemic affected how they provided T2DM care.

**Table 1:** Comparison of provider characteristics for high vs. low GLP‐1RA and SGLT‐2i prescribers.

Characteristic	High‐prescribing providers, *n* = 9	Low‐prescribing providers, *n* = 8
Median years of experience (IQR)	7 (3.5, 16)	17.5 (11.5, 23)
Median number of patient‐facing hours per week (IQR)	25 (23, 28)	21.5 (13, 40)
Learned GLP‐1RAs and SGLT‐2is in residency training (number of +/− indicates how many providers said yes/no)	+ + + − − − − − −	− − − − − − − −
Common specialties (# of providers)	Internal medicine‐pediatrics (4) Family medicine (3) Internal medicine (2)	Geriatrics (3) Internal medicine (3) Family medicine (1) Internal medicine‐pediatrics (1)

In contrast to many high‐prescribing providers, many low‐prescribing providers stated that their training did not include prescribing GLP‐1RA or SGLT‐2i. In general, internal medicine–pediatrics was a frequently cited high‐prescribing specialty, whereas geriatrics was a low‐prescribing specialty. Due to this distinction in patient age groups, high‐prescribing providers were more concerned about how side effects could interfere with their patients′ responsibilities. However, elderly patients tend to have dexterity issues and other medical complications, so low‐prescribing providers were more worried about route of administration and reducing polypharmacy. These approaches, divided by primary care type, can be found in Supporting Information 1.

From the interview transcripts, four common themes emerged: access to healthcare, prescription costs and insurance processes, provider collaboration, and medication novelty. Table 2 notes the shared barriers and facilitators of GLP‐1RA and SGLT‐2i prescription mentioned by all providers whereas Table 3 separates these themes based on the differences between high and low‐prescribing provider responses. Provider‐provided solutions and their associated implementation strategies are included in Table 4.

**Table 2:** Common barriers and facilitators across all providers of GLP‐1RA/SGLT‐2i prescriptions.

Barriers	Facilitators
Patients struggle to access healthcare (lack of transportation, Internet, and provider availability)	Outcome visibility
Patient mistrust of new therapies and misinformation/lack of knowledge	Trusting relationship between patient and provider (relationship longevity)
Cost/insurance	Task‐sharing (pharmacist for medication education/teaching and prior authorization team)
Prescription inertia (lack of collaboration in medication management between specialists and primary care)	Patient familiarity with new medications (Ozempic is well known by patients before discussing it with them as part of individual clinical care)

**Table 3:** High vs. low GLP‐1RA and SGLT‐2i prescribing provider barriers and facilitators.

TICD domain	Barrier or facilitator	High providers	Low providers	Quotes
Incentives and resources	Cost mitigation	Willing to navigate in‐system resources	Cost issues seem insurmountable; unsustainable options	“It’s a lot of work, but we have several patients, though, who we were able to get them [GLP‐1RAs] through the patient assistance program.”—High provider 8
				“I am completely pessimistic that we are ever going to get more support or more financial resources for these medications. It’s just wasted breath.”—Low provider 1
Incentives and resources	Insurance processes	Effective in‐clinic prior authorization teams	Poor prior authorization due to centralization and overwork	“We have a prior auth team now that helps, you know, get the medicines approved or denied without the burden on the clinics as much as it used to be.”—High provider 4
				“We used to do prior for these medications on site at our clinic. And then they centralized it. And the centralized version just does not work that well…They do not spend the time like looking through the patient’s chart. They just route everything back to you a zillion times.”—Low provider 2
Professional interactions	Support	Strong reliance on embedded pharmacist	Opts for specialist referrals or search engines	“I get most of my information from the pharmacist. She sends me updated information on comparisons between the different medications, what the hemoglobin A1c reductions are”—High provider 13
				“I’ve never done this, but if I had a question, in theory, I would do probably an E‐consult just to run it by them [a specialist]. If I have questions about the medications, I’ll usually use UpToDate or ChatGPT”—Low provider 15
Individual health professional factors	Treatment approach	Metformin and GLP‐1RAs co–first line	Emphasis on lifestyle modification first	“I actually prefer a GLP‐1RA to Metformin, depending on what else they have. If somebody comes in and they have obesity, even if their A1c is 6.5, and they are open to being on an injection medication, I will pretty much always do a GLP‐1RA first.”—High provider 14
				“The first thing is always exploring lifestyle, which is probably better than any medication we are going to prescribe.”—Low provider 9
Patient factors	Major concerns	Side effects and patient preference	Polypharmacy and route of administration	“First, I talk to the patient about what they might prefer and make sure they understand the side effects. I have some patients, for example, with the SGLT‐2is that do not want to even try them if they already have issues with urinary symptoms or recurrent yeast infections.”—High provider 5
				“These medications add to the pill burden, and so we have to justify why we have to utilize this. If they have multiple reasons to be on this medication, that way, we are checking off multiple boxes with one pill.”—Low provider 3

**Table 4:** Barriers mapped to ERIC implementation strategies.

Barriers	Proposed solutions	Strategy
Lack of trained staff	“I feel like we need PharmD people or PharmD training people who are highly able to do this [prior authorizations] efficiently.”—Low provider 17	Recruit, designate, and train for leadership
Lack of newer medication awareness in patients	“Maybe doing like some sort of patient outreach for patients to learn about the newer medications…We [physicians] will just refill the medications, whereas when patients ask me, I’m more likely to like, really think about it.”—Low provider 17	Conduct educational meetings
Medication costs and insurance gaps	“I think the health system can invest more on programs that could subsidize costs for patients, especially patients who are either not fully covered by insurance, or have high co‐pays, or are in this concept of do not hold.”—Low provider 3	Use other payment schemes
Time restrictions in appointments leading to unanswered questions	“Having easier follow up could definitely be one change. Somebody gets nervous about a new diabetes diagnosis, or is a little bit scared, and it then is taking them 6 to 8 weeks to come in to get in a clinic visit, and they are nervous for 6 to 8 weeks to get that information.”—High provider 14	Revise professional roles
Patient misinformation and expectations	“They could develop a series of simple audience, friendly layman termed delivered speeches or presentations about some of these newer medicines, so that the patient is not so surprised when they go in to see their primary care doc.”—Low provider 1	Develop educational materials
Access to support services and providers	“I like the idea of co‐locating other services in real time, and that can range from things like food but also specialists. So, if a patient could come to a multidisciplinary center and be able to, you know, potentially see a few providers at once that would be really nice.”—High provider 4	Promote network weaving
Medication unfamiliarity in providers	“I recently went to a conference, and there was some like extra special tips on how to prescribe GLP1s if they are not covered by insurance. Just lots of different extending the dosing interval or click counting. I still need to learn more about all of this stuff. But yeah, just like best practices, or like helpful tips and tricks could be exchanged more.”—High provider 12	Conduct ongoing training, capture and share local knowledge, and audit and provide feedback

### 3.1. Access to Healthcare

Entry to and participation in healthcare was a barrier to T2DM care, including the prescription of medications such as GLP‐1RAs and SGLT‐2is, and was mentioned in every provider interview. Due to a shortage of PCPs, patients encountered variable wait times for appointments. Once scheduled, providers described additional factors that impact engagement in T2DM care such as securing reliable transportation for appointments or making the time to arrive early to appointments. Additional outer contextual factors that influence T2DM care and treatment include the ability to engage in telehealth, which requires stable Internet connection and technological prowess. Although a particular hospital may be lauded for its world‐class care, one provider explained:

“Access is the biggest problem in the …system. I always say quality of care is important …but I personally feel that access should come first and then quality, because patients at least need to be seen first.”*—*High provider 6

Although a couple of providers witnessed more SDOH in patients from racial or ethnic minorities, the consensus was that access disparities occurred along socioeconomic lines. Specifically, lower income patients—such as retired patients with fixed incomes—struggled with access to healthy foods and could not adhere to diet plans designed to help reduce hemoglobin A1c. Furthermore, GLP‐1RAs must be refrigerated, which can be difficult for individuals experiencing housing instability. Typically, many patients do not encounter only one barrier when seeking healthcare. The compounding nature of SDOH was illustrated in an example given by one provider:

“Social determinants often stack on the patients and families that are struggling with medication, access, housing, transportation. I think those are kind of all like an accordion.”—High provider 4

To address access issues, providers recommended offering multidisciplinary appointments to reduce travel and colocating resources. For example, one provider stated that healthcare systems could partner with local organizations to set up in‐hospital food pantries. In addition, patients would not have to take multiple workdays off if they could see their specialists on the same day. The same provider outlined a successful strategy implemented at his part‐time clinic location:

“I work a half day at an underserved youth clinic …We have a very robust—we call it the store—but it′s got a huge amount of healthy foods, and it′s available 5 days a week for patients to come and access food, personal care, products, books, and clothes, but our UofM clinic doesn′t have that even though it is more of a clinic that patients who have some of the social determinants are often routed.”—High provider 4

### 3.2. Prescription Costs and Insurance Processes

Another barrier expressed by PCPs was the burden of GLP‐1RA and SGLT‐2i out‐of‐pocket costs. However, high and low‐prescribing providers varied in how they navigated this challenge. High prescribers were more likely to have knowledge of funding opportunities and be more proactive in directing patients to financial resources available within the health system. In contrast, several low prescribers viewed prescription costs as insurmountable and offered unsustainable solutions. For example, one provider advised his patients to order their prescriptions from Canada to offset prices. Regardless of the approach, most providers called for healthcare leadership to invest in more extensive subsidized support programs. According to one PCP,

“There are a lot of patients in this situation called, ‘Do Not Hold’, where insurance is hardly covering anything anymore…They have to decide between having a heater service paid for versus buying this new medication.”—Low provider 3

Even though a few high‐prescribing providers mentioned less insurance restraints compared with the past, coverage under high‐deductible plans remains often partial, insufficient, and unclear. These individual nuances were noted as providers and their staff found it confusing to navigate approvals for GLP‐1RA and SGLT‐2i therapies. Furthermore, for many patients, their insurance coverage of GLP‐1RAs or SGLT‐2is only applies once less expensive medications like metformin or sulfonylureas prove to be ineffective or intolerable on their own. Providers stressed that understanding how much a patient is restricted by their plan and providing all the necessary paperwork for prior authorizations requires lengthy, frustrating back‐and‐forth between providers and insurance companies. They emphasized the need for trained prior authorization personnel embedded within their practices as the stress of navigating insurance guidelines was evident in the words of one PCP:

“When I think about it [GLP‐1RAs or SGLT‐2is], or a patient asks about it, I kind of get like a visceral reaction like, ‘Oh, God, I′m going to have to go through this circus of what does your insurance cover?’”—Low provider 17. Additional quotes can be found in Table 3.

For high‐prescribing providers, the time‐consuming process of navigating insurance requirements and approvals is handled by other staff members like social workers, pharmacists, or teams specially created for prior authorizations. However, the majority of low‐prescribing providers noted that they submitted prior authorizations on their own or with very little support. Thus, potential strategies to address this issue could be recruiting more staff or revising the responsibilities of current staff to handle such processes and reduce stress on PCPs.

### 3.3. Provider Collaboration

Besides prior authorizations, embedded pharmacists fulfill additional responsibilities as physician‐extenders. Because of the newness of GLP‐1RAs and SGLT‐2is, high‐prescribing providers turned to their pharmacists for questions on side effects and equivalent dosage. Pharmacists also conducted follow‐up phone calls with patients. Such task‐sharing is effective as it allows patients to express concerns that cannot be addressed during the limited duration of a primary care appointment. If a patient experiences adverse effects and does not know whether they should continue taking a medication, getting ahold of the pharmacist is much faster than a specialist. Conversely, low‐prescribing providers usually relied more on specialists and search engines for prescription inquiries due to the structure of their clinics and colleague accessibility. They lacked in‐clinic pharmacists or found the information in online search engines such as UpToDate adequate.

Although task‐sharing was a shared facilitator among all providers, it can also act as a barrier when members of the care team fail to take initiative. Instead of starting a GLP‐1RA or an SGLT‐2i for a shared patient, specialty physicians such as endocrinologists and cardiologists will often direct patients to ask their PCPs. This hesitancy fosters treatment delays and concentrates pressure on PCPs, especially for resource‐deprived PCPs who lack prior authorization staff compared to specialists who are equipped with the dedicated resources. One provider expressed her exasperation with prescription inertia from subspecialists:

“I′m like, well, you′re a doctor, too. You can start it [the medication]. I understand it′s complicated, but you know, you have one organ, and I have, like all of them…I can′t do it all.”—Low provider 17

### 3.4. Medication Novelty

Low‐prescribing providers tended to have graduated residency earlier than high prescribers. Due to their recent introduction, many low‐prescribing providers stated that their training did not include GLP‐1RAs or SGLT‐2is. Implementing tools like dashboards, EHR prompts, and order sets to review all eligible patients can also help to ensure that GLP‐1RAs and SGLT‐2is are offered across the populations. Gaps between training and practice can be addressed by having additional people involved such as pharmacists to consult/review, as well as emphasizing information dissemination. During the interviews, every PCP highlighted the necessity of engaging in conferences, continuing medical education (CME), and organizational meetings to stay updated. One provider explained the need to conduct ongoing training and emphasized the benefits of attendance:

“I recently went to a conference, and there was some like extra special tips on how to prescribe GLP1s if they′re not covered by insurance. Just lots of different extending the dosing interval or click counting. I still need to learn more about all of this stuff. But yeah, just like best practices, or like helpful tips and tricks could be exchanged more.”—High provider 12

Additionally, both patients and providers alike have scrutinized the adoption of these new medications. Although they proved to drastically reduce hemoglobin A1c, some providers conveyed concerns over the uncertainty of long‐term effects. Without this data, they occasionally found it difficult to convince patients to take the leap of faith. On the patient′s end, much of their skepticism stemmed from a mistrust in pharmaceutical companies, which was especially evident among racial and ethnic minorities who feared being subjects of an “experimental” therapy. Despite personal beliefs held by patients, PCPs were still able to build rapport due to the provider–patient relationship longevity that comes with practicing in primary care. This longitudinal relationship was described by one provider:

“Patients tend to have a relationship with their primary care that is beyond anything else I get with any specialist. You become part of their family in many ways.”—Low provider 3

In addition to healthcare providers, patients were reported to have external sources of trusted information such as family members and social media. Many arrive at their appointments already familiar with GLP‐1RAs or SGLT‐2is because their loved one had a prescription. Furthermore, GLP‐1RAs like semaglutide became popularized by advertisements and celebrity endorsements as a successful weight loss agent. The role word‐of‐mouth plays in the acceptance of GLP‐1RAs and SGLT‐2is was depicted by one provider:

“Patients come to me asking for these medications, and I think some of that is related to the success stories people are hearing from the news or what visible outcomes people are seeing from their family members that were overweight for such a long time.”—High provider 10

Although the presence of GLP‐1RAs and SGLT‐2is in the media does aid in convincing patients, not all information a patient receives is correct. At times, misinformation can lead to unrealistic patient expectations as some may believe that taking the medication without lifestyle modification will still result in rapid health improvement. Therefore, providers recommended that healthcare systems develop and disseminate more patient‐facing educational materials. In this way, patients can consider any questions before their appointments. If they had no prior awareness of GLP‐1RAs or SGLT‐2is, they can also better advocate for themselves and start the conversation with their providers. Furthermore, more clinic time could be spent reviewing other comorbidities or preventative health measures like cancer screenings and vaccinations.

## 4. Discussion

Qualitative analysis of PCP interviews revealed the barriers and facilitators of prescribing newer and highly effective GLP‐1RA and SGLT‐2i medications for T2DM. Four major themes emerged from the transcripts as influencing prescribing patterns: access to healthcare, prescription costs and insurance processes, provider collaboration, and medication novelty. High‐prescribing providers were typically more proactive in navigating prescription costs and insurance issues with support from their colleagues. Additionally, the recent introduction of these medications presented challenges, especially for low prescribers who were not exposed to GLP‐1RAs and SLGT‐2is in training.

In a Michigan Medicine study analyzing a database of national health insurance claims from 2009 to 2018, Type 1 diabetics were found to incur the most in total and out‐of‐pocket costs, followed by Type 2 diabetics, then individuals without diabetes. On average, Type 2 diabetics accumulated $14,220 in total expenses and $1,122 in out‐of‐pocket expenses—over half of which were attributed to prescriptions [15]. Currently, there are no generic brands for GLP‐1RAs and SGLT‐2is, thus complicating the search for cost‐effective solutions. However, some patients may opt to obtain “knock‐offs” at compound pharmacies, especially during shortages, but this method is often criticized for providing non–FDA‐approved medications [16]. For those with sufficient income, self‐pay options through pharmaceutical companies do offer price cuts by removing third‐party supply chain fees. These programs are intended to allow easier access for patients without insurance or patients whose insurance coverage does not extend to these medications. Although such discounted options cannot assist low‐income individuals who may need it most, as monthly out‐of‐pocket prescription costs can remain as high as several hundred dollars.

Additionally, there was a clear difference in the resources offered to low‐prescribing providers, which resulted in a heavier cognitive burden as they were to balance clinic appointments with insurance paperwork and searching for creative financial solutions. Although treatment approach is also influenced by personal philosophy and patient preference, lack of staff support was a major barrier in a provider′s ability to initiate prescriptions. Most high‐prescribing providers opted for metformin and a GLP‐1RA co–first line, whereas more low‐prescribing providers elected for lifestyle modification and metformin, which may be partially motivated by the urge to circumnavigate more complex prior authorizations. Such shifts in prescribing patterns due to prior authorizations were similarly reported by the American Academy of Allergy, Asthma, and Immunology [17].

Outside of T2DM, GLP‐1RAs and SGLT‐2is have been approved for the management of heart disease, MASLD, obstructive sleep apnea, and chronic kidney disease [18, 19]. Their efficacy is also being studied in other conditions, including alcohol use disorder [20]. Due to their various applications, subspecialists becoming accustomed to prescribing GLP‐1RAs and SLGT‐2is could aid in offering therapies while also unburdening PCPs in comanaging patients with complex, chronic diseases. It is estimated that clinical inertia affects 30%–60% of diabetic patients and results in missed opportunities to enhance the long‐term benefits of early blood sugar management [21]. Half of clinical inertia causes are attributed to health professional‐level factors such as competing priorities and unfamiliarity with new medications [21]. Medical audits of health records can improve prescribing habits. In fact, half of the high‐prescribing providers interviewed in this study noted that their clinics audited based on hemoglobin A1c management.

Considering the newness of these medications, provider and patient education is crucial. Providers suggested designing standardized educational supplements and disseminating them through the patient portal. A systematic review of patient education through patient portals determined that the majority of available educational materials were utilized. Patients found them useful, and they positively impacted health outcomes [22]. As for providers, the issue of treatment familiarity will resolve itself as younger doctors enter healthcare, but CME participation will remain salient as we continue to learn more about GLP‐1RAs and SGLT‐2is. Another method to consider is provider–patient aligned education. In a study where providers attended the 2021 Metabolic and Endocrine Disease Summit and patients learned about GLP‐1RAs from texts and videos matched to symposium education, providers reported more confidence in GLP‐1RA prescription, and patients reported a strong intention to ask their PCPs about starting a GLP‐1RA posteducation [23].

There are several strengths and limitations of this study. This is the first qualitative study aimed at understanding provider‐level barriers and facilitators of GLP‐1RA and SLGT‐2i prescription in T2DM patients. Secondly, the use of CFIR allowed identification of factors across several key domains, which then enabled selection of implementation strategies. Furthermore, this study includes a diverse set of providers from varying primary care types, positions of leadership, and locations of practice. One limitation of this study is that all low prescribers should not necessarily be high prescribers as certain patient populations cannot be safely or reasonably prescribed GLP‐1RAs or SGLT‐2is. The focus of this study is to highlight factors that are associated with high and low GLP‐1RA and SGLT‐2i usage and to promote thought around system‐level changes that can better support providers. The prescribing rates were also crudely calculated, which may not account for all the complexities encountered in clinical practice such as pregnancy and women of childbearing age. Another potential limitation is the sample size of PCPs interviewed, although no new themes emerged. Additionally, participating PCPs were sourced from the same health system, thus making generalizability to other settings challenging. Finally, due to evolving guidelines, coverage changes, and growing provider familiarity, prescribing patterns for GLP‐1RAs and SGLT‐2is may have changed between 2021, when the rates were analyzed, and 2025, when the interviews were conducted—thus resulting in a possible misclassification of low providers.

## 5. Conclusions

By utilizing an approach informed by implementation frameworks, we qualitatively examined barriers and facilitators of GLP‐1RA and SGLT‐2i primary care prescribing patterns in a tertiary health care setting. All providers noted patient‐centered barriers rooted in socioeconomic stability such as prescription costs, insurance restrictions, and healthcare access. Meanwhile, task‐sharing, medication popularity, and patient–provider rapport appeared as shared facilitators in overcoming patient mistrust and care management. Provider‐proposed solutions were mapped to implementation strategies that may help inform future studies that can address pharmcoinequities and improve the prescription of newer, highly effective treatments in patients with T2DM.

**Nomenclature**
MASLD: metabolic dysfunction‐associated steatotic liver disease
T2DM: Type 2 diabetes‐mellitus
GLP‐1RA: glucagon‐like peptide‐1 receptor agonists,
SGLT‐2i: sodium/glucose cotransporter 2 inhibitor

## Author Contributions

Writing—original draft preparation: P.W. with support from V.L.C and P.V.P.; conceptualization: V.L.C. and P.V.P.; data collection and analysis: P.W. and P.V.P.

## Funding

This work was supported by the Michigan Center for Diabetes Translational Research, P30DK092926.

## Ethics Statement

All research activities were approved and reviewed by the University of Michigan Institutional Review Board. Informed consent was captured on recording prior to interview initiation in accordance with the Declaration of Helsinki.

## Conflicts of Interest

The authors declare no conflicts of interest.

## Supporting information


**Supporting Information** Additional supporting information can be found online in the Supporting Information section. **Supporting Information 1:** is a table of key approaches to Type 2 diabetes management across primary care types. **Supporting Information 2:** is the provider interview guide with the Consolidated Framework for Implementation Research (CFIR) domains in brackets.

## Data Availability

The authors confirm that the data supporting the findings of this study are available within the article.
