# Supplementary material for: Identifying Provider Prescribing Practices for GLP‐1RAs and SGLT‐2is in Patients With Type 2 Diabetes to Address Pharmacoinequity
Source: J Diabetes Res. 2026 Feb 26;2026:7439681. doi: 10.1155/jdr/7439681 (PMC12943470; doi:10.1155/jdr/7439681)
Supplement: Supplementary file 1 — Supporting Information Additional supporting information can be found online in the Supporting Information section. Supporting Information 1: is a table of key approaches to Type 2 diabetes management across primary care types. Supporting Information 2: is the provider interview guide with the Consolidated Framework for Implementation Research (CFIR) domains in brackets. [file JDR-2026-7439681-s001.docx]

**Supplement 1: Key Approaches to T2DM Management Across Primary Care Types**

| Internal Medicine (6 providers) | Family Medicine (4 providers) | Internal medicine-pediatrics  (4 providers) | Geriatrics (3 providers) |
| --- | --- | --- | --- |
| Start with a patient’s history w/ diabetes: how much do they know, what meds have they tried, how long since diagnosis, where are they on their diet and exercise journey | Treatment approach dependent on diagnosis phase (new vs returning patient), A1c, weight, and comorbidities | More aggressive on younger patients or those with multiple comorbidities that can tolerate a lower goal  For older patients, focus on addressing symptoms/side effects, balancing risks/benefits, and avoiding polypharmacy | First and foremost, considers patient age, then comorbidities and refers patient for education  Concerns over polypharmacy and Medicare restrictions/fixed income |
| Considers patients preference (values, goals, route of administration, side effects) | Considers patients preference (values, goals, route of administration, side effects) | Considers patients preference (values, goals, route of administration, side effects) | Considers patients preference (values, goals, route of administration, side effects) |
| Metformin and GLP-1RAs co first-line | Metformin first-line | Metformin and GLP-1RAs co first-line | Metformin first-line |
| Less of a team approach | Integrates physician-extenders (LPNs, MAs, pharmacists, dietitians, social work) | Integrates physician-extenders (LPNs, MAs, pharmacists, dietitians, social work) | Multidisciplinary practice |
| Education through national and local conferences, UptoDate, webinars, journals | Education through department grand rounds | Education through department grand rounds and journals | Education through national conferences |

**Supplement 2: Provider Interview Guide with CFIR Domains in Brackets**

**General Role in Michigan Medicine [Characteristics of Individuals]**

1. Please describe your general role at Michigan Medicine.

**Current type 2 diabetes mellitus Approach [Characteristics of Individuals]**

1. How do you work with patients with type 2 diabetes mellitus in your practice? What is your approach?
   1. After you identify a patient with type 2 diabetes mellitus, what do you typically do?
   2. What resources and/or treatments do you offer?
   3. How do you decide what resources and/or treatments to offer (e.g., severity of diabetes, medical comorbidities, pregnancy status, past diabetes treatment history, personal support)?

**Current Approach to Prescribing with glucagon-like peptide receptor agonists (GLP-1RAs) and sodium/glucose cotransporter 2 inhibitors (SGLT-2is) in Type 2 Diabetes Mellitus [Innovation]**

1. Who else do you think needs to be involved in type 2 diabetes mellitus treatment (e.g., other clinicians, family members, who else on the team handles prior authorizations etc.)?
   1. What are your concerns when treating type 2 diabetes mellitus patients with glucagon-like peptide receptor agonists (GLP-1RAs) and sodium/glucose cotransporter 2 inhibitors (SGLT-2is)?
   2. What gets in the way of providing glucagon-like peptide receptor agonists (GLP-1RAs) and sodium/glucose cotransporter 2 inhibitors (SGLT-2is) (i.e., patient education, prescribing restrictions, pharmacy involvement)?
      1. Do you ever have conversations with patients about what makes glucagon-like peptide receptor agonists (GLP-1RAs) and sodium/glucose cotransporter 2 inhibitors (SGLT-2is) treatment challenging? (e.g., reasons for declining or not following through with treatment?)
   3. What has made you feel successful in treating patients with type 2 diabetes mellitus with glucagon-like peptide receptor agonists (GLP-1RAs) and sodium/glucose cotransporter 2 inhibitors (SGLT-2is) treatment? What has been hard about treating patients?

**Current glucagon-like peptide receptor agonists (GLP-1RAs) and sodium/glucose cotransporter 2 inhibitors (SGLT-2is) prescribing approach [Inner Setting]**

1. What do you think other providers in your clinic are doing with type 2 diabetes mellitus patients who meet criteria for consideration of glucagon-like peptide receptor agonists (GLP-1RAs) and sodium/glucose cotransporter 2 inhibitors (SGLT-2is)? Do you talk with other providers to see how they care for patients with type 2 diabetes mellitus?
   1. What kinds of information and materials about glucagon-like peptide receptor agonists (GLP-1RAs) and sodium/glucose cotransporter 2 inhibitors (SGLT-2is) have been made available to you? (*e.g., websites, trainings, conferences, please try to get specifics*)
   2. Who do you ask if you have questions about glucagon-like peptide receptor agonists (GLP-1RAs) and sodium/glucose cotransporter 2 inhibitors (SGLT-2is) management? What information have you asked about?

**Current glucagon-like peptide receptor agonists (GLP-1RAs) and sodium/glucose cotransporter 2 inhibitors (SGLT-2is) Prescribing Approach [Outer Setting]**

1. Where do you think patients with type 2 diabetes mellitus should be treated with glucagon-like peptide receptor agonists (GLP-1RAs) and sodium/glucose cotransporter 2 inhibitors (SGLT-2is)? (e.g., primary care, endocrine?)
2. How did the COVID pandemic change how you provide type 2 diabetes mellitus care? Did the pandemic made it harder or easier to provide type 2 diabetes mellitus related care? In what ways? (*Discuss telehealth vs. in-person care*)

**Health Equity [Outer Setting]**

1. Do you see unique barriers at your site for your patients because of race, ethnicity, or gender? If yes, how so? (*if the participant has trouble answering, probe about the following one at a time*: What about differences in reliable transportation, distance from medical center, access to childcare, stable housing, variable working hours?)
   1. What do you think your clinic/hospital could be doing better to engage patients with type 2 diabetes mellitus with glucagon-like peptide receptor agonists (GLP-1RAs) and sodium/glucose cotransporter 2 inhibitors (SGLT-2is) treatment in the groups you mentioned who may have barriers?

**Health Equity [Innovation]**

1. When you meet with patients from historically marginalized groups, such as women or patients of color, how do you change or tailor type 2 diabetes mellitus care?

**Health Equity [Inner Setting]**

1. What do you think your clinic does especially well when it comes to prescribing type 2 diabetes mellitus medications to patients group of color in conversation about initiating glucagon-like peptide receptor agonists (GLP-1RAs) and sodium/glucose cotransporter 2 inhibitors (SGLT-2is)?
2. Has your team or clinic ever collected or looked at data related to equitable type 2 diabetes mellitus care?
   1. What kinds of equity-related trainings occur, or are available, at your clinic?

**Improving type 2 diabetes mellitus glucagon-like peptide receptor agonists (GLP-1RAs) and sodium/glucose cotransporter 2 inhibitors (SGLT-2is) treatment [Inner Setting, Outer Setting, Individuals – iterative coding based on responses/strategies]**

1. If your clinic were to do more to address type 2 diabetes mellitus glucagon-like peptide receptor agonists (GLP-1RAs) and sodium/glucose cotransporter 2 inhibitors (SGLT-2is) treatment needs for patients, what would be needed? (*Probe for strategies, examples may include – hiring more staff, increased time in clinical encounters, more training in evidence-based care, better/consistent leadership support, audit & feedback, clinic champions, help from endocrine providers, larger systemic changes at the hospital-level*)
   1. What would make shared decision-making a more common clinical approach towards encouraging patients with type 2 diabetes mellitus to seek glucagon-like peptide receptor agonists (GLP-1RAs) and sodium/glucose cotransporter 2 inhibitors (SGLT-2is) treatment at your clinic?
2. Do you think your clinic leadership would be supportive of doing more to address type 2 diabetes mellitus glucagon-like peptide receptor agonists (GLP-1Ras) and sodium/glucose cotransporter 2 inhibitors (SGLT-2is) treatment needs?
